# Visceral and subcutaneous abdominal adiposity and pulmonary function in 30-year-old adults: a cross-sectional analysis nested in a birth cohort

**DOI:** 10.1186/s12890-017-0510-7

**Published:** 2017-11-28

**Authors:** Paula Duarte de Oliveira, Fernando César Wehrmeister, Bernardo Lessa Horta, Rogelio Pérez-Padilla, Giovanny Vinícius Araújo de França, Denise P. Gigante, Fernando C. Barros, Ken K. Ong, Emanuella De Lucia Rolfe, Ana Maria Baptista Menezes

**Affiliations:** 1 Federal University of Pelotas - Postgraduate Program in Epidemiology, Rua Marechal Deodoro, 1160, 3° andar, Pelotas, RS Zip code: 96020-220 Brazil; 2 National Institute of Respiratory Diseases, Calzada De Tlalpan, 4502 Mexico City, DF Mexico; 3 Ministry of Health - Secretariat of Health Surveillance, Esplanada dos Ministérios Bloco G, Brasilia, DF Zip code: 70058-900 Brazil; 4 Catholic University of Pelotas - Postgraduate Program in Health and Behavior, Rua Gonçalves Chaves, 373, Pelotas, RS Zip code: 96015-560 Brazil; 5 Medical Research Council (MRC) Epidemiology Unit, Institute of Metabolic Science, University of Cambridge School of Clinical Medicine, Cambridge Biomedical Campus, Cambridge, UK

**Keywords:** Pulmonary function, Body composition, Abdominal adiposity, Visceral adiposity

## Abstract

**Background:**

Several studies have verified body fat distribution in association with pulmonary function (PF), mainly waist circumference, but few have used measures able to distinguish abdominal fat compartments. The present study aims to verify the association of visceral adipose tissue (VAT) and subcutaneous adipose tissue (SAT) with PF measures.

**Methods:**

In 1982, all hospital births occurring in Pelotas, Brazil, were identified and those livebirths have been followed. In 2012–13, the cohort participants were evaluated and VAT and SAT measured using ultrasound; forced expiratory volume in the first second (FEV_1_) or forced vital capacity (FVC) were patronized in z-scores stratified by sex. The associations were verified using crude and adjusted linear regressions.

**Results:**

The present analyses comprised 3438 individuals (1721 women). VAT was inversely associated with spirometric parameters, in both crude and adjusted models. SAT showed inverse associations in the crude analyzes in males and a positive trend after adjustment, except for SAT and FVC in males. To each centimeter of VAT, mean adjusted FEV_1_ z-scores decreased 0.072 (95% CI -0.107; −0.036) in men and 0.127 (95% CI -0.164; −0.090) in women, and FVC z-scores decreased −0.075 (95% CI -0.111; −0.039) and 0.121 (95% CI -0.158; −0.083), in men and women, respectively.

**Conclusions:**

VAT has a consistent inverse association with FEV_1_ and FVC in both sexes. On the other hand, SAT showed inconsistent results with PF parameters.

**Electronic supplementary material:**

The online version of this article (10.1186/s12890-017-0510-7) contains supplementary material, which is available to authorized users.

## Background

Many studies have shown negative influences of obesity on pulmonary function (PF) in the last decades [1]. These findings have been attributed to the load imposed by the adipose tissue on the ventilatory mechanics [2–4], or to an adverse impact of obesity on the respiratory control, and more recently to systemic inflammation caused by excess of fat that could lead to airway inflammation and subsequent airflow obstruction [4]. However, most of these studies measured body mass index, which is incapable to distinguish adipose tissue from other body components [5]. In addition, the upper body fat mass seems to play a more important role on PF impairment [1, 4, 6], and measures such as waist circumference (WC), even having a high correlation with abdominal fat [5], cannot differentiate visceral adipose tissue (VAT) from subcutaneous adipose tissue (SAT).

Few studies [3, 7–11] with conflicting results, have utilized devices capable to separate abdominal VAT and SAT to verify its association with PF, such as computed tomography (CT) or ultrasound. CT is the gold-standard device, but demands high costs and radiation risks and it is unfeasible in many research scenarios. As a more accessible device, abdominal ultrasound has demonstrated precise results on adipose tissue measures, presenting a good correlation with CT measures (*r* = 0.68–0.74) [5, 7]. To our knowledge, ultrasound was used in only two previous studies on this issue, in specific women samples [8, 9].

The present study aims to verify the association of VAT and SAT, measured by abdominal ultrasound, with spirometric function in a young adult population that have been prospectively followed since birth.

## Methods

In 1982, the maternity hospitals in Pelotas, southern Brazil, were visited daily and the deliveries were identified. Those liveborns whose family lived in the urban part of the city were examined and their mothers interviewed. These subjects have been prospectively followed. In 2012–13, we tried to contact the cohort members, who were invited to visit the study clinic. After signing the informed consent form, the subjects were interviewed, examined and donated a blood sample. The 30 years follow-up project was approved by the Federal University of Pelotas Ethics Committee under protocol 16/12. More details on the methodology of the cohort study are described in previous publication [12].

For the purposes of the current report, a cross-sectional analysis was performed using data from all cohort participants who had spirometry information at 30 years follow-up. The exclusion criteria for spirometry were: mental problems not allowing the understanding of the testing procedures, the self report of pregnancy, active tuberculosis, heart disease, recent surgery (thoracic, abdominal, or ocular in the previous 3 months), and recent retinal detachment (3 months). The spirometric variables analyzed were the Forced Expiratory Volume in the first second (FEV_1_) and the Forced Vital Capacity (FVC) prior to the bronchodilator use. Both were assessed with a portable ultrasonic spirometer (EasyOne, Ndd Medical Technologies Inc., Zürich, Switzerland). We followed the procedures recommended by the American Thoracic Society/European Respiratory Society [13], aiming for three acceptable maneuvers with a maximum variation of 150 mL between the two highest values.

**Fig. 1:**
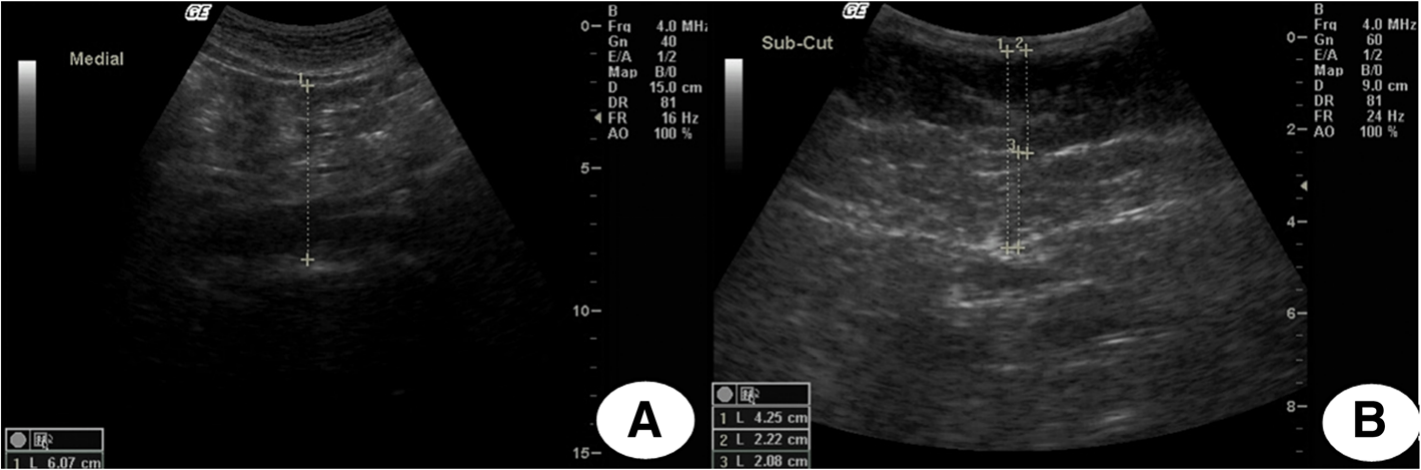
Ultrasound image examples to abdominal adipose tissue measurement. **a** visceral adipose tissue thickness. **b** subcutaneous adipose tissue thickness and components (deep and superficial)

In the 2012–13 visit, we also assessed subcutaneous abdominal adipose tissue (SAT) and visceral adipose tissue (VAT) thicknesses using an abdominal ultrasound (Toshiba Xario, Toshiba Medical Systems Corp, Tokyo, Japan). The probe was placed at the crossing point between the xyphoid line and the waist circumference using electronic callipers. SAT was defined as the distance on the sagittal plane between posterior line of dermis to the outer bowel wall and VAT as the distance from the peritoneum boundary to the lumbar spine (in millimeters) [14, 15] Two different static images were obtained at the end of a quiet expiration by applying minimal pressure, ensuring no displacement of the abdominal cavity. As previously described [16] three trained technicians performed the ultrasound scans using a standardized protocol, and all measurements were performed immediately after obtaining each image (Figure 1). Quality control procedures were conducted during the data collection. Quality control sessions were carried out in order to estimate the intra and inter-observer technical errors by comparing the results obtained by each of the three technicians to those of one investigator (GVAF) who had been previously trained and certified in this technique. Three sessions were conducted before starting data collection, with 10 participants each. The relative inter-observer technical error of measurement was 3.1% for both measures, while the relative intra-observer technical error of measurement for VAT was 4.1% and 3.4% for SAT, as described in previous publication [17]. This data were collected by two trained radiology technicians.

The analyses were performed using software Stata version 12.2 (Stata Corp., College Station, TX, USA). The variables were described using mean and standard deviation for continuous variables and absolute and relative frequencies for categorical variables. Also, the correlations between the continuous variables (PF and body variables) were verified. The associations between VAT or SAT and FEV_1_ or FVC were evaluated using linear regressions and, due to the significant interaction between sex and abdominal fat variables, analyses were stratified by sex. The linearity of the associations was verified using fractional polynomial regressions (results not shown in the tables), indicating that the linear regression model was not different from the best model fitted with exponentials. FEV_1_ and FVC results were expressed as z-scores, which were generated from the standardized residuals of the study sample, taking into account skin color and height, stratified by sex.

In the multivariate model birth weight (in grams), maternal smoking during pregnancy, actual height (measured by stadiometer, in centimeters), total fat mass (percentage in relation to total mass, measured using air displacement plethysmography, BOD POD® Composition System; COSMED, Albano Laziale, Italy), weight (in kilograms, measured by the BOD POD scale), skin color (self-reported in white, black, brown and others), schooling (complete years in formal education), family socioeconomic level (National Economic Index [18]), smoking at 30 years (never smoker, former smoker, smoker), self-reported wheezing in last year, any kind of corticosteroid use in the last 3 months and habitual physical activity (minutes spent in leisure-time activities per week), were included. All variables were collected at 30 years follow-up visit (except birth weight and maternal smoking during pregnancy) and selected for the adjusted model, a priori, by statistical and/or theoretical criteria (see Additional file 1: Table S1). Also, VAT and SAT were included simultaneously in the model. The variation inflation factor was verified to ensure the absence of collinearity between the model variables (variation inflation factor < 10) and the only variable removed from the adjusted analyses was WC. Values of *p* < 0.05 in the Wald’s test for linear tendency were considered statistically significant.

## Results

In 2012–13, 3701 subjects were evaluated, which added to the 325 known to have died, represented a follow-up rate of 68.1%. Data on spirometry was available for 3438 individuals (1721 women) who were included in the present report. The quality criteria according to the American Thoracic Society/European Respiratory Society guidelines [13] were met in 91.6% of the exams. The characteristics of the individuals included in the analyses were similar to those included in the initial cohort regarding sex and socioeconomic level (data not shown in tables), ensuring general population representativeness.

Table 1 shows the description of the sample regarding perinatal, socioeconomic, demographic, behavior, body composition and PF characteristics, stratified by sex. Most of the individuals were white (75.1% and 76.4%, men and women, respectively) and had 12 or more years of schooling (39.0% and 48.8%, men and women, respectively). Most of the sample had never smoked; 13.3% of men and 16.5% of women reported wheezing episodes in the previous year to the interview. Women had higher total fat mass compared to men (37.3 versus 24.5%); on the other hand, men had higher mean VAT than women (6.9 versus 4.9 cm). Concerning the spirometry, men had mean FEV_1_ and FVC 1.1 L and 1.3 L, respectively, higher than women. Exposure to smoking during intrauterine life and skin color were the only variables without significant statistical difference between sexes.

**Table 1:** Description of the sample as covariates, nutritional status, abdominal adiposity and lung function. 1982 Pelotas Birth Cohort (*n* = 3438)

Social, demographic, behavioral variables and nutritional status	Male (*n* = 1717) N (%)	Female (*n* = 1721) N (%)	*p*
Birth weight (grams)^a^			0.003
> = 2500	1614 (94.0)	1572 (91.4)	
<2500	103 (6.0)	148 (8.6)	
Maternal smoking during pregnancy^a^			0.932
No	1115 (64.9)	1120 (65.1)	
Yes	602 (35.1)	601 (34.9)	
Skin color			0.121
White	1290 (75.1)	1315 (76.4)	
Black	274 (16.0)	274 (15.9)	
Brown	95 (5.5)	80 (4.7)	
Others	58 (3.4)	52 (3.0)	
Education (years)			< 0.001
0–4	103 (6.1)	102 (6.0)	
5–8	381 (22.4)	290 (17.0)	
9–11	551 (32.5)	481 (28.2)	
≥ 12	662 (39.0)	833 (48.8)	
Smoking status			0.008
Never	978 (57.0)	1040 (60.5)	
Former	296 (17.2)	312 (18.2)	
Smoker	443 (25.8)	366 (21.3)	
Wheezing in the last year			0.009
No	1488 (86.7)	1437 (83.5)	
Yes	229 (13.3)	284 (16.5)	
Use of corticoids in the last three months^b^			< 0.001
No	1576 (95.5)	1480 (89.9)	
Yes	75 (4.5)	167 (10.1)	
Physical activity in leisure^c^			< 0.001
Inactive	1040 (61.7)	1341 (79.0)	
Active	646 (38.3)	356 (21.0)	
Nutritional status (body mass index)			< 0.001
Underweight	25 (1.5)	44 (2.6)	
Normal weight	610 (35.7)	772 (45.0)	
Overweight	697 (40.7)	493 (28.7)	
Obese	379 (22.2)	407 (23.7)	
Anthropometric, adiposity and pulmonary function variables	Mean (SD)	Mean (SD)	P*
Height (cm)	174.4 (6.9)	161.4 (6.2)	< 0.001
Weight (kg)	82.2 (16.8)	69.6 (16.2)	< 0.001
Waist circumference	89.2 (11.8)	80.6 (12.0)	< 0.001
Total fat mass (%)	24.5 (9.2)	37.3 (8.5)	< 0.001
Subcutaneous adipose tissue (cm)	1.9 (1.0)	2.6 (1.2)	< 0.001
Visceral adipose tissue (cm)	6.9 (2.0)	4.9 (1.7)	< 0.001
FEV_1_ (liters)	4.0 (0.6)	2.9 (0.5)	< 0.001
FVC (liters)	4.8 (0.8)	3.5 (0.5)	< 0.001

*N* number of observations, *SD* standard deviation, *BMI* body mass index, *FEV*_*1*_ forced expiratory volume in the first second, *FVC* forced vital capacity

^a^Variables collected in the perinatal follow up, other variables at 30 years

^b^Maximum number of missing values: 104 observations in corticoids use

^c^Cutoff point for physical activity - 150 min/week as recommended by the World Health Organization for adults. *P*-value by qui-squared test *p-value by t-test

The correlations between VAT, SAT, PF and other body variables are shown in Table 2. It can be observed that the two abdominal fat variables have a lower correlation between them compared with other body fat variables.

**Table 2:**
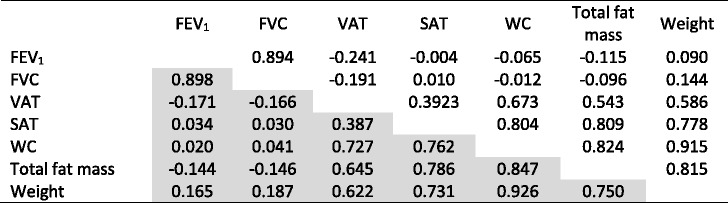
Correlation between body composition variables and pulmonary function, stratified by sex

Grey cells – males correlations. *FEV*_*1*_ forced expiratory volume in the first second, *FVC* forced vital capacity, *SAT* subcutaneous adipose tissue, *VAT* visceral adipose tissue, *WC* waist circumference

**Fig. 2:**
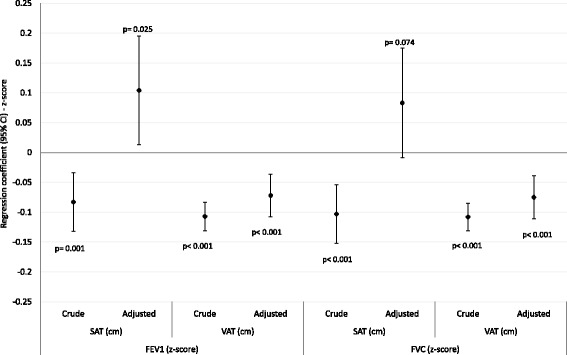
Linear regressions: spirometric measures (z-scores) and abdominal fat, males (*n* = 1701). SAT: subcutaneous adipose tissue; VAT: visceral adipose tissue; FEV1: forced expiratory volume in the first second; FVC: forced vital capacity; CI: confidence interval; Adjusted by subcutaneous or visceral fat, weight, total fat mass (%), asset index, scholarship (complete years), smoking status (never, former, active smoker), self-reported wheezing in the last year, corticoids use in the last three months, physical activity, birth weight and maternal smoking during pregnancy. *P*-values: Wald’s test for linear tendency

**Fig. 3:**
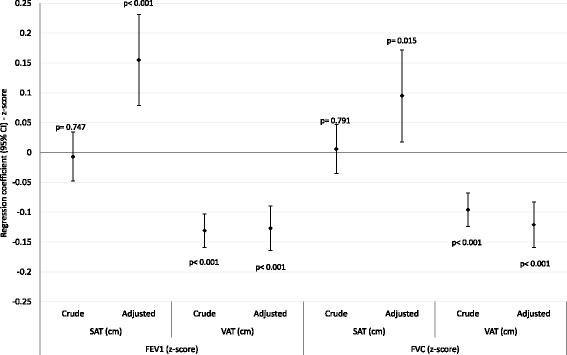
Linear regressions: spirometric measures (z-scores) and abdominal fat, females (*n* = 1712). SAT: subcutaneous adipose tissue; VAT: visceral adipose tissue; FEV1: forced expiratory volume in the first second; FVC: forced vital capacity; CI: confidence interval; Adjusted by subcutaneous or visceral fat, weight, total fat mass (%), asset index, scholarship (complete years), smoking status (never, former, active smoker), self-reported wheezing in the last year, corticoids use in the last three months, physical activity, birth weight and maternal smoking during pregnancy. *P*-values: Wald’s test for linear tendency

Figures 2 and 3 shows the associations between VAT, SAT and PF parameters. We observed the same VAT trends for both sexes: consistent inverse association with FEV_1_ and FVC, in both crude and adjusted models. Regarding the adjusted analyzes for the several confounders, for each additional centimeter of VAT, mean adjusted FEV_1_ z-scores decreased 0.072 (95% CI -0.107; −0.036) in men and 0.127 (95% CI -0.164; −0.090) in women, and FVC z-scores decreased −0.075 (95% CI -0.111; −0.039) and 0.121 (95% CI -0.158; −0.083), in men and women, respectively. These z-score results represent, in absolute values, mean FEV_1_ decrease of 43 mL and 64 mL and FVC 60 mL and 61 mL, in men and women, respectively, for each centimeter increment of VAT.

SAT showed different patterns of association with PF. Regarding the crude analyzes, we can observe inverse associations only among men. On the other hand, after adjustment, men and women showed positive associations between SAT and both PF parameters (except for SAT and FVC in men, but in the same trend - Figs. 2 and 3).

## Discussion

The main result to be highlighted is that increases in VAT were strong predictors of reduced spirometric measures, in both sexes, more importantly in women. In order to explore the effects of each abdominal fat compartment independently, multivariate models including VAT and SAT were utilized, since correlation between them was low (*r* = 0.39 for men and women). As mentioned previously, measures such as WC are more accessible, but cannot differentiate abdominal compartments, which is the main objective of this study. WC and PF results, as other body measures in this same population, can be found in previous publication [19]; the present findings are in accordance with other previous studies that had investigated central obesity by different measure methods and research settings [20, 21].

Similar results using CT to assess abdominal fat were observed by Park et al. among Korean individuals aged 15 to 85 years [11]: in women, an increase in VAT was associated with reduced PF, while in men the best predictor of reduced lung function was total abdominal adipose tissue, VAT and SAT, after adjustment for height, weight, age, WC, systolic blood pressure and inflammatory markers, not using VAT and SAT in the same model.

We used ultrasound measurements of abdominal as proxies for these abdominal fat masses. While the validity of ultrasound in this specific setting has not yet been determined, some validation studies using the same standardized protocol have found strong correlations between ultrasound and magnetic resonance imaging estimates of abdominal fat in a variety of settings and populations [14, 22, 23]. A strict quality control process was carried out and we could identify consistent sex differences in the distribution of these abdominal fat compartments compared with previous reports using other imaging methods [14, 22, 24–27].

The role of the adipose tissue is not limited to lipids deposit. There is also evidence of its role as endocrine organ, producing a number of pro-inflammatory molecules and cytokines such as inteleuncine-6 and C-reactive protein, the last found in high levels in the ones with high VAT accumulation and highly related to cardiovascular episodes [28, 29]; VAT has been also known as insulin resistant compartment [30]. On the other hand, SAT has controversial findings regarding its role on the diseases risk. In non-caucasian populations this compartment showed associations with several metabolic changes [31–33], but in recent studies it showed a protective effect and lack of association in obese individuals [34, 35].

The two main hypotheses for PF reduction due to excess of abdominal fat are via inflammatory mediators [4] and/or by mechanical restriction [2–4]. Whether the reduced lung function is due to one or the other cannot be completely clarified in our study, we can just have clues that the mechanical restriction seems to be more important, since reduction of both parameters, FEV_1_ and FVC, were observed.

VAT has been associated to several cardiometabolic risk factors, metabolic syndrome and systemic inflammation [30]. However, two previous studies [3, 11] investigated abdominal fat compartments using CT and reduced lung function, but neither of them supported the inflammation hypothesis: no association was found between inflammatory markers and PF [3] or persistence of the association between abdominal adiposity with lower PF after controlling for some inflammatory makers in the adjusted statistical model [11].

SAT showed an inverse association with PF in previous publications [3, 8, 11]. In our study, we observed positive associations between SAT and PF after adjustment and this result must be interpreted carefully due to the lack of plausibility that SAT increment can be beneficial to PF. In our population, the mean SAT is still low at 30 years old and could lead to an absence of influence on reduction of PF parameters. Also, SAT accumulation has been associated with a normal metabolic profile, while VAT accumulation, considered ectopic, is associated with an altered metabolic profile due to habits such as smoking and low physical activity [30]. This might explain this SAT positive trend, mainly after adjustment for VAT and total fat mass. Only one study we are aware of [10], using CT in an elderly population, did not find association concerning VAT, SAT and PF, although they had find an inverse association between PF and central obesity measured by dual-energy X-ray absorptiometry.

Some strengths of our study should be mentioned. We used a large general population sample, with information for several confounding factors. This allows us power to detect the associations and extrapolate results to similar healthy populations. Also we had rigorous quality control in spirometry and ultrasound measures, a method that allows a good estimation of adiposity compartments (VAT and SAT) despite the literature on adiposity measured by US and FP is scarce.

On the other hand, we did not perform any static lung volume measures, such as total lung capacity or expiratory reserve volume; we have just spirometric measurements, more accessible in this research scenario than methods based on plethysmography or inert gas dilution. Although spirometric and traditional anthropometric measurements were performed several times during cohort follow-ups, abdominal ultrasound measurements were carried out only at the 30th year follow-up allowing just cross-sectional analysis between abdominal fat compartments and PF outcomes preventing us to infer causality.

## Conclusion

In this 30-years-old general population sample, VAT showed an inverse association with PF reduction, with consistent results between crude and adjusted analysis, FEV_1_ and FVC and both sexes, collaborating to the board of evidences and alerts about the organism damages associated to this kind of ectopic fat accumulation. On the other hand, SAT does not show a clear association pattern and can be considered a poor predictor of lung function in this population, showing agreement with the health lifestyle hypothesis linked with subcutaneous fat deposition.

## Additional file

Additional file 1: Table S1. Association between pulmonary function (z-score) and confounding variables. 1982 Pelotas Birth Cohort (*n* = 3438). (DOCX 58 kb)

## Abbreviations

CT: Computed tomography
FEV_1_: Forced expiratory volume in the first second
FVC: Forced vital capacity
PF: Pulmonary function
SAT: Subcutaneous adipose tissue
VAT: Visceral adipose tissue
WC: Waist circumference
