# Supplementary material for: Visceral and subcutaneous abdominal adiposity and pulmonary function in 30-year-old adults: a cross-sectional analysis nested in a birth cohort
Source: BMC Pulm Med. 2017 Nov 28;17:157. doi: 10.1186/s12890-017-0510-7 (PMC5704528; doi:10.1186/s12890-017-0510-7)
Supplement: Additional file 1: Table S1. — Association between pulmonary function (z-score) and confounding variables. 1982 Pelotas Birth Cohort (n = 3438). (DOCX 58 kb) [file 12890_2017_510_MOESM1_ESM.docx]

**Additional Information**

**Visceral and subcutaneous abdominal adiposity and pulmonary function in 30-year-old adults: a cross-sectional analysis nested in a birth cohort.**

Supplementary Table 1. Association between pulmonary function (z-score) and confounding variables. 1982 Pelotas Birth Cohort (n= 3,438).

|  | Males (n= 1,717) | | Females (n= 1,721) | |
| --- | --- | --- | --- | --- |
| Confounding variables | FEV_1_ (z-score)  β (CI 95%) | FVC (z-score)  β (CI 95%) | FEV_1_ (z-score)  β (CI 95%) | FVC (z-score)  β (CI 95%) |
| Asset index (quintiles) | p= 0.758 | p= 0.946 | p< 0.001 | p= 0.049 |
| 1 (poorest) | - | - | - | - |
| 2 | -0.009 (-0.157; 0.140) | 0.004 (-0.145; 0.153) | 0.194 (0.053; 0.336) | 0.156 (0.015; 0.297) |
| 3 | 0.040 (-0.100; 0.180) | 0.054 (-0.086; 0.194) | 0.266 (0.132; 0.401) | 0.190 (0.056; 0.324) |
| 4 | 0.020 (-0.169; 0.209) | 0.029 (-0.160; 0.219) | 0.235 (0.054; 0.416) | 0.161 (-0.020; 0.342) |
| 5 (richest) | 0.084 (-0.066; 0.234) | 0.022 (-0.129; 0.173) | 0.245 (0.100; 0.390) | 0.168 (0.023; 0.313) |
| Education (years) | p= 0.077 | p< 0.001 | p= 0.071 | p< 0.001 |
| 0 - 4 | - | - | - | - |
| 5 - 8 | 0.128 (-0.090; 0.346) | 0.197 (-0.020; 0.415) | 0.189 (-0.035; 0.413) | 0.279 (0.054; 0.503) |
| 9 - 11 | 0.157 (-0.053; 0.367) | 0.144 (-0.067; 0.354) | 0.337 (0.125; 0.549) | 0.258 (0.046; 0.471) |
| ≥ 12 | 0.238 (0.031; 0.446) | 0.246 (0.039; 0.454) | 0.440 (0.236; 0.644) | 0.351 (0.146; 0.556) |
| Smoking status* | p= 0.444 | p= 0.189 | p< 0.001 | p= 0.005 |
| Never | - | - | - | - |
| Ex-smoker | -0.054 (-0.184; 0.076) | 0.048 (-0.082; 0.178) | 0.078 (-0.048; 0.204) | 0.199 (0.073; 0.325) |
| Smoker | -0.067 (-0.180; 0.045) | 0.104 (-0.009; 0.217) | -0.227 (-0.346; -0.108) | -0.017 (-0.136; 0.103) |
| Wheezing in the last year* | p= 0.006 | p= 0.536 | p< 0.001 | p= 0.467 |
| No | - | - | - | - |
| Yes | -0.195 (-0.335; -0.056) | 0.044 (-0.096; 0.184) | -0.366 (-0.492; -0.239) | -0.047 (-0.175; 0.080) |
| Corticoid use in the last three months* | p= 0.931 | p= 0.641 | p= 0.056 | p= 0.607 |
| No | - | - | - | - |
| Yes | -0.010 (-0.241; 0.221) | 0.055 (-0.176; 0.285) | -0.157 (-0.318; 0.004) | 0.042 (-0.119; 0.203) |
| Physical activity in leisure* | p= 0.046 | p= 0.271 | p= 0.159 | p= 0.124 |
| Inactive | - | - | - | - |
| Active | 0.100 (0.002; 0.198) | 0.055 (-0.043; 0.153) | 0.084 (-0.033; 0.201) | 0.092 (-0.025; 0.208) |
| Fat mass (quintiles) | p< 0.001 | p< 0.001 | p< 0.001 | p= 0.002 |
| 1^th^ (lowest) | - | - | - | - |
| 2^nd^ | -0.001 (-0.146; 0.145) | 0.030 (-0.115; 0.175) | -0.200 (-0.348; -0.052) | -0.131 (-0.279; 0.018) |
| 3^rd^ | -0.177 (-0.323; -0.031) | -0.115 (-0.262; 0.030) | -0.278 (-0.428; -0.128) | -0.141 (-0.292; 0.009) |
| 4^th^ | -0.294 (-0.439; -0.148) | -0.305 (-0.451; -0.161) | -0.266 (-0.415; -0.117) | -0.150 (-0.299; -0.001) |
| 5^th^ (highest) | -0.626 (-0.772; -0.479) | -0.656 (-0.802; -0.509) | -0.415 (-0.565; -0.265) | -0.320 (-0.471; -0.169) |
| Birth weight (grams)* | p= 0.187 | p= 0.846 | p= 0.446 | p= 0.473 |
| >=2500g | - | - | - | - |
| <2500g | -0.134 (-0.334; 0.065) | -0.019 (-0.219; 0.179) | -0.065 (-0.234; 0.103) | -0.061 (-0.230; 0.107) |
| Maternal smoking during pregnancy* | p= 0.249 | p= 0.858 | p= 0.194 | p= 0.166 |
| No | - | - | - | - |
| Yes | -0.058 (-0.158; 0.041) | 0.009 (-0.090; 0.108) | 0.065 (-0.034; 0.165) | 0.091 (-0.008; 0.190) |

FEV_1_: forced expiratory volume in the first second; FVC: forced vital capacity; β: regression coefficient; CI: confidence interval; p-values by Wald’s test of linear tendency, except *p-values by Wald’s test of heterogeneity.
